# Trends and determinants of excess winter mortality in New Zealand: 1980 to 2000

**DOI:** 10.1186/1471-2458-7-263

**Published:** 2007-09-24

**Authors:** Gabrielle S Davie, Michael G Baker, Simon Hales, John B Carlin

**Affiliations:** 1 Injury Prevention Research Unit, Dunedin School of Medicine, University of Otago, PO Box 913, Dunedin, New Zealand; 2 Department of Public Health, Wellington School of Medicine, University of Otago, Wellington, New Zealand; 3 School of Population Health, University of Melbourne, and Clinical Epidemiology and Biostatistics Unit, Murdoch Childrens Research Institute, Melbourne, Australia

## Abstract

**Background:**

Although many countries experience an increase in mortality during winter, the magnitude of this increase varies considerably, suggesting that some winter excess may be avoidable. Conflicting evidence has been presented on the role of gender, region and deprivation. Little has been published on the magnitude of excess winter mortality (EWM) in New Zealand (NZ) and other Southern Hemisphere countries.

**Methods:**

Monthly mortality rates per 100,000 population were calculated from routinely collected national mortality data for 1980 to 2000. Generalised negative binomial regression models were used to compare mortality rates between winter (June–September) and the warmer months (October–May).

**Results:**

From 1980–2000 around 1600 excess winter deaths occurred each year with winter mortality rates 18% higher than expected from non-winter rates. Patterns of EWM by age group showed the young and the elderly to be particularly vulnerable. After adjusting for all major covariates, the winter:non-winter mortality rate ratio from 1996–2000 in females was 9% higher than in males. Mortality caused by diseases of the circulatory system accounted for 47% of all excess winter deaths from 1996–2000 with mortality from diseases of the respiratory system accounting for 31%. There was no evidence to suggest that patterns of EWM differed by ethnicity, region or local-area based deprivation level. No decline in seasonal mortality was evident over the two decades.

**Conclusion:**

EWM in NZ is substantial and at the upper end of the range observed internationally. Interventions to reduce EWM are important, but the surprising lack of variation in EWM by ethnicity, region and deprivation, provides little guidance for how such mortality can be reduced.

## Background

Seasonal fluctuations in mortality have been documented since 400BC [1]. Although many countries currently experience an increase in mortality during winter, the magnitude of this increase varies considerably, suggesting that some winter excess may be avoidable. Paradoxically, relative seasonal variation in mortality appears lowest in countries with cold winters, such as Russia, Norway and Canada and is higher in Britain, Israel and Portugal where winters are milder [2-5]. In Britain around 40,000 more deaths occur in the winter months than expected from the non-winter mortality rates [6].

Infant mortality displays the characteristic winter high and summer low [7]. Seasonal mortality is greatest in the elderly but British data indicate it is not confined to this age group, with one study reporting that the chance of dying in winter exceeded that in the rest of the year by more than 10% in the 46–64 years age group [4].

Evidence for a gender difference in winter mortality is inconsistent [4,6]. Limited research has been conducted on the association between seasonal variation in mortality and ethnicity [7]. Although the relationship between overall mortality and social deprivation is unquestionable, the extent to which socioeconomic factors impact on seasonal excess mortality is unclear. In a cross-country analysis, strong relationships were found between EWM and relative income poverty, inequality, deprivation and fuel poverty [8]. In Great Britain from 1986 to 1996, there was little association between deprivation and EWM for all cause mortality although lack of central heating was associated with increased seasonal mortality [9].

Localised climates experienced by different geographical regions within a country do not consistently lead to differences in extent of seasonality of mortality by region [4,6]. EWM from 1976–1987 for ten standard regions of England and Wales displayed little variation whereas Swedish mortality data from 1974–1984 indicated a strong regional association between cold exposure and high coronary mortality [4,10].

Following the recent decline in influenza epidemics, about half of all excess cold-related mortality can be attributed to Ischaemic Heart Disease (IHD) and Cerebrovascular Disease (CVD) mortality. Respiratory disease (RD) mortality accounts for nearly half of the remaining excess [11-13]. In a number of countries including the Netherlands and Germany, a decline in seasonal mortality has been observed over the last century [14,15].

Research into seasonal mortality in NZ is limited and has mainly focused on particular causes of mortality. Using national mortality data in over 35 year olds from 1970–83, seasonal aspects of coronary heart disease were reported with peak winter (mid-July) to summer variation estimated to be 35% [16]. Mortality from 1980–1984 indicated the presence of major seasonal variation by month in coronary heart disease and CVD mortality for males, females and both islands of NZ [17].

The objective was to describe the size and distribution of seasonal mortality in NZ, in particular the patterns of EWM by age, gender, ethnicity, region and deprivation. Also of interest was identifying causes of death with high EWM.

## Methods

### Mortality data

Routinely collected mortality data were obtained from the New Zealand Health Information Service (NZHIS), Ministry of Health, for all deaths registered from 1 January 1980 to 31 December 2001. As mortality data are provided according to registration date, deaths occurring outside of this period were excluded. Due to expected under-representation of mortality in 2001 due to late registration, mortality that occurred after 31 January 2001 was not included.

If an individual's ethnic group was recorded as Maori, ethnicity was assumed to be Maori; all remaining individuals were categorised as Non-Maori. Four Regional Health Authorities (RHAs) were used for the geographical analysis. The NZDep ordinal scale, which is based on 9 census variables that reflect aspects of material and social deprivation, was used to provide small-area level deprivation scores [18]. NZDep96, available for all of NZ's 1700 Census Area Units (CAUs), assigns a score of 1 to the decile of census areas with the least deprivation and 10 to CAUs with the most deprived scores. Cause of death was examined based on ICD-10 chapter classification.

### Population denominators

Population denominators, obtained from Statistics New Zealand (SNZ), were counts from the 5-yearly censuses and estimates for the non-census years. Population estimates stratified by age group, gender, ethnicity, DHB and NZDep for the 1996 and 2001 censuses were also obtained from SNZ.

### Methods of data analysis

Monthly mortality rates per 100,000 were calculated with all months standardised to 30-days with adjustment for leap years. In line with research in the Northern Hemisphere, four-month seasons were used with winter in NZ defined as June to September. [8,19] Absolute EWM refers to the difference between the four-month winter mortality and the average mortality in the preceding summer/autumn (in NZ, February–May) and successive spring/summer (in NZ, October–January); relative EWM is the ratio of these. The relative winter:non-winter EWM less one gives the percentage that the observed winter deaths is above that which is expected from the non-winter deaths. This has previously been referred to as the Coefficient of Seasonal Variation in Mortality (CSVM) and the Excess Winter Death Index (EWDI). [4,8]

We used regression models for rates in order to obtain inferences for mortality rate ratios (MRRs) assessing the extent to which mortality rates in winter differed from non-winter. As goodness-of-fit tests indicated that a Poisson distribution was not adequate for modelling the process generating the mortality counts, MRRs were estimated using a generalised negative binomial regression model. This model is a version of the negative binomial model in which the overdispersion parameter can be modelled as a function of other variables, allowing a much improved fit to data such as these [20].

The count of mortality events was the outcome variable, person-years divided into winter and non-winter periods relative to the number of winter and non-winter days in each year reflected the population exposed and a binary winter indicator (0 = non-winter, 1 = winter) was included as the explanatory variable. Inclusion of age (6 categories; 0–4, 5–14, 15–29, 30–59, 60–79, 80+ years), gender, ethnicity (Maori or non-Maori), region (RHA level), NZDep and the interactions of these with the winter indicator in the regression model enabled analysis of the extent to which particular population subgroups had different EWM rates. Because of inconsistent definitions of ethnicity and changing regional boundaries over time, the full model including age, gender, ethnicity, region and NZDep was restricted to mortality events that occurred in the most recent 5-year period for which data were available. Using a four month winter period and restricting to the winters of 1996–2000, the dataset included all deaths occurring between 1 February 1996 and 31 January 2001.

Subsets based on cause of death were analysed using the same regression model.

The percentage of EWM attributable to particular causes of death was calculated by obtaining the difference between the winter and non-winter mortality rates per 100,000 person-years for each ICD-10 chapter and dividing by the total excess which was calculated from the sum of differences that indicated EWM had occurred.

Stata version 8.2 was used for all statistical analysis [21].

## Results

### Seasonal mortality; 1980–2000

**Figure 1:**
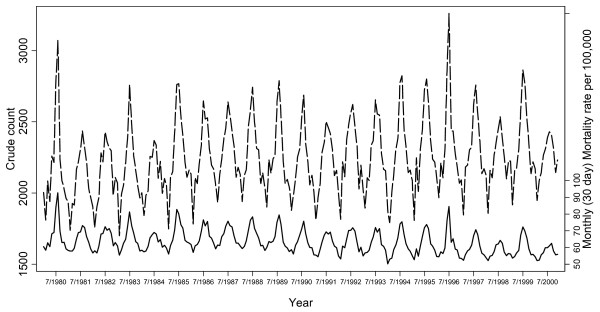
Monthly variation in crude mortality and monthly (30-day) mortality rates per 100,000 from January 1980 to December 2000.

For the 21-year period from 1 February 1980 to 31 January 2001, there were 565,308 registered deaths. Monthly frequencies indicate strongly that seasonal mortality does occur in NZ (Figure 1). Mortality was typically highest in July and lowest in February with on average, 600 more deaths occurring in July than in February. Annual peaks in mortality varied considerably in magnitude. In comparison with the lowest monthly mortality rate recorded in January 1994, the 3075 deaths that occurred in August 1980 produced a mortality rate that was almost twice as large.

Absolute EWM over this period ranged from 818 in 1984 to 2388 in 1996. The absolute EWM rate was highest in 1980 when there were 71 excess winter deaths per 100,000 and lowest in 2000 when 21 excess winter deaths per 100,000 occurred. Over the 21-year period, an average of almost 1600 excess winter deaths occurred each year at a rate of 45 per 100,000.

**Figure 2:**
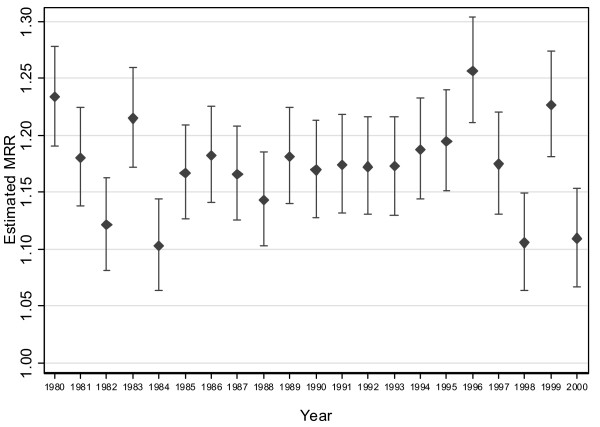
Annual variation in estimated winter:non-winter all-cause mortality rate ratios from 1980 to 2000.

The estimated winter:non-winter all-cause MRRs for each year from 1980 to 2000 indicate that relative seasonal EWM was clearly greater in some years than others (Figure 2). Mortality during winter 1996 was 25.6% (95% confidence interval (CI) 21.1% to 30.4%) higher than expected, the largest observed over this 21 year period. The winter of 1984 had the lowest estimated MRR with winter mortality 10.3%, (95% CI 6.3% to 14.4%) higher than expected. There was no evidence for a secular trend in EWM over this 21-year period (p = 0.9). Over this 21 year period, mortality was estimated to be 18.4% (95% CI 5.9% to 32.4%) higher during winter than expected.

### Detailed analysis of seasonal mortality; 1996–2000

From 1 February 1996 to 31 January 2001 there were 137,583 registered deaths in NZ with gender, age group, prioritised ethnicity, region and NZDep recorded for 95.7%. Total all-cause mortality during winter occurred at a rate of 8.0 per 1000 person years over this 5-year period; 14% higher than expected from non-winter rates. Unadjusted winter:non-winter ratios indicate winter mortality was higher than expected in those aged sixty years or older (Table 1).

**Table 1:** Rates of all-cause mortality in winter and non-winter months, ratio of rates and relative change in winter:non-winter ratios for potential modifying factors; 1996–2000

	Rate per 1000 person years (No of deaths)*		**Winter:non-winter ratio (95% CI)‡ Unadjusted**	**Winter:non-winter ratio relative to baseline category§ (95% CI)‡**	**p-value**
	**Winter**	**Non-winter**			

**All**	8.0 (49232)	6.7 (82427)	1.14 (0.98, 1.33)		

**Age (years):**					
0–4	1.7 (772)	1.5 (1407)	1.12 (0.91, 1.38)	1.00	
5–14	0.2 (174)	0.2 (376)	0.93 (0.75, 1.17)	0.84 (0.68, 1.03)	
15–29	0.9 (1120)	0.9 (2240)	1.01 (0.79, 1.27)	0.91 (0.75, 1.09)	
30–59	2.5 (6188)	2.4 (11572)	1.04 (0.86, 1.26)	0.96 (0.84, 1.10)	
60–79	25.1 (20123)	21.2 (33915)	1.15 (0.98, 1.35)	1.05 (0.92, 1.20)	
80+	122.8 (20855)	97.2 (32917)	1.23 (1.11, 1.36)	1.10 (0.92, 1.31)	p = .09
**Gender:**					
Male	8.3 (24882)	7.1 (42628)	1.10 (0.91, 1.34)	1.00	
Female	7.8 (24350)	6.4 (39799)	1.20 (0.99, 1.46)	1.09 (1.01, 1.19)	p = .04
**Ethnicity**†:					
Non-Maori	7.7 (44583)	6.4 (74116)	1.19 (1.02, 1.40)	1.00	
Maori	13.2 (4649)	11.3 (8311)	1.13 (0.94, 1.34)	1.01 (0.91, 1.10)	p = .92
**NZDep96:**					
1–3	6.9 (11341)	5.7 (18869)	1.11 (0.85, 1.44)	1.00	
4–6	7.9 (14693)	6.7 (24689)	1.14 (0.89, 1.46)	0.97 (0.87, 1.08)	
7–10	8.8 (23198)	7.4 (38869)	1.16 (0.91, 1.18)	0.97 (0.87, 1.08)	p = .78
**RHA:**					
North	7.1 (15039)	6.0 (25479)	1.23 (0.92, 1.65)	1.00	
Midland	8.2 (10040)	7.0 (16925)	1.08 (0.80, 1.45)	0.97 (0.87, 1.10)	
Central	8.3 (12478)	6.9 (20498)	1.18 (0.88, 1.58)	1.06 (0.94, 1.19)	
South	8.9 (11675)	7.5 (19525)	1.08 (0.80, 1.46)	1.01 (0.89, 1.14)	p = .55

* No of deaths in winter correspond to a 4 month period; non-winter, an 8 month period

‡ Generalised negative binomial regression model; overdispersion a linear combination of age group and ethnicity

§ Adjusted for all other covariates including covariate by winter interaction terms

† Mortality rates have been age-standardised to account for different population structures of Maori and Non-Maori

The exponentiated coefficients of the winter-by-explanatory-variable interaction terms from the generalised negative binomial regression model are ratios of the winter:non-winter MRRs relative to a baseline category of the explanatory variable. These estimates can be interpreted as "relative risks" of excess winter deaths compared with the baseline. After adjusting for all other covariates, estimates of the magnitude of winter:non-winter mortality suggested higher mortality in under 5-year olds than in those aged 5–59. Mortality in those aged eighty or over appeared most affected by winter with an estimated 10% higher incidence in winter than non-winter relative to that in the under-fives. After adjusting for all other covariates in the model it was estimated that females were 9% more likely to die in winter than males. There was no evidence to suggest that winter:non-winter MRRs differed by ethnicity, NZDep or RHA. A three-way interaction term that allowed winter:non-winter MRRs to differ by gender across age groups provided insufficient statistical evidence (p = 0.6) to warrant inclusion in the final model.

Mortality in the 5–14 age category was relatively rare with only 550 (0.4%) deaths over the five years. Combination of this age group with the under-fives age group produced similar results indicating analysis using 6 age groups was insensitive to the relatively few deaths in this age group.

Mortality from diseases of the respiratory system was most dependent on seasonal effects; with a winter mortality rate that was 83%, (95% CI 71%, 96%) higher than expected from the non-winter rates (Table 2). Mortality from diseases of the blood and blood-forming organs and certain disorders involving the immune mechanism, endocrine, nutritional and metabolic diseases, diseases of the circulatory system and diseases of the digestive system were estimated to occur between 20%–26% more often in winter than in non-winter. Symptoms, signs and abnormal clinical and laboratory findings, not elsewhere classified caused mortality in winter around 47% more often in winter than in non-winter. For certain infectious and parasitic diseases, the corresponding estimate was 68%. Six percent of EWM was attributable to mortality from neoplasms, 3.6% from diseases of the blood and blood-forming organs and certain disorders involving the immune mechanism and 3.3% from endocrine, nutritional and metabolic diseases. Seasonal mortality attributable to each of the remaining causes was less than 2%.

**Table 2:** Rates of death in winter and non-winter months, ratio of rates and relative change in winter:non-winter ratios for all causes of death; 1996–2000

		Rate per 1000 person years (No of deaths)*		**Winter:non-winter ratio (95% CI)§**
		**Winter**	**Non-winter**	
**ICD 10 Chapter‡**				
I	Infection	5.4 (332)	3.4 (422)	1.68 (1.38, 2.05)
II	Neoplasms	214.9 (13184)	206.7 (25297)	1.03 (0.98, 1.08)
III	Blood etc.	18.7 (1150)	14.0 (1719)	1.26 (1.11, 1.43)
IV	Endocrine etc.	28.3 (7733)	23.8 (2916)	1.25 (1.12, 1.39)
VI	Mental	4.4 (271)	4.9 (602)	0.91 (0.70, 1.18)
VI	Nervous	16.6 (1017)	14.0 (1716)	1.11 (0.96, 1.27)
VII	Eye	0.0 (0)	0.0 (2)	
VIII	Ear	0.1 (5)	0.1 (13)	0.75 (0.21, 2.72)
IX	Circulatory	350.2 (21482)	287.5 (35189)	1.20 (1.15, 1.24)
X	Respiratory	97.9 (6005)	57.4 (7027)	1.83 (1.71, 1.96)
XI	Digestive	21.5 (1319)	18.4 (2247)	1.25 (1.11, 1.40)
XII	Skin	1.9 (114)	1.5 (184)	1.29 (0.97, 1.71)
XIII	Musculoskeletal	6.1 (375)	5.1 (626)	1.14 (0.94, 1.39)
XIV	Genitourinary	10.9 (667)	9.5 (1167)	1.11 (0.96, 1.29)
XV	Pregnancy	0.1 (4)	0.1 (15)	0.87 (0.25, 3.03)
XVI	Perinatal	3.6 (223)	3.6 (436)	0.95 (0.76, 1.19)
XVII	Congenital	5.4 (333)	4.9 (605)	1.15 (0.94, 1.41)
XVIII	Symptoms	4.2 (256)	3.1 (381)	1.47 (1.20, 1.81)
XIX	Injury	45.0 (2760)	47.3 (5789)	0.93 (0.91, 1.08)

* No of deaths in winter correspond to a 4 month period; non-winter, an 8 month period

‡ I: Certain infectious and parasitic diseases; II: Neoplasms; III: Diseases of the blood and blood-forming organs and certain disorders involving the immune mechanism; III Diseases of the blood and blood-forming organs and certain disorders involving the immune mechanism; IV: Endocrine, nutritional and metabolic diseases; V: Mental and Behavioural disorders; VI: Diseases of the nervous system; VII: Diseases of the eye and adnexa; VIII: Diseases of the ear and mastoid process; IX: Diseases of the circulatory system; X: Diseases of the respiratory system; XI: Diseases of the digestive system; XII: Diseases of the skin and subcutaneous tissue; XIII: Diseases of the musculoskeletal system and connective tissue; XIV: Diseases of the genitourinary system; XV: Pregnancy, childbirth and the puerperium; XVI: Certain conditions originating in the perinatal period; XVII: Congenital malformations, deformations and chromosomal abnormalities; XVIII: Symptoms, signs and abnormal clinical and laboratory findings, not elsewhere classified; XIX: Injury, poisoning and certain other consequences of external causes.

§ Generalised negative binomial regression model; overdispersion a linear combination of year

Mortality caused by diseases of the circulatory system accounted for 47% of all excess winter deaths over this 5-year period with mortality from diseases of the respiratory system accounting for 31%. The mortality rate for diseases of the respiratory system during winter was 9.4 per 10,000 person years over this 5-year period; 66% higher than expected from non-winter rates (Table 3). For under-five year olds, mortality associated with respiratory disease was estimated to be almost 2.5 times higher in winter than expected. From the age of 15, increasing age was associated with increasing winter:non-winter respiratory disease MRRs with those aged eighty or older 66% more likely to die in winter than expected. The winter excess of respiratory mortality was slightly higher for females than males (p = 0.08) but there was no evidence to suggest that winter:non-winter mortality differs by age, ethnicity, region or deprivation.

**Table 3:** Rates of mortality from diseases of the respiratory system in winter and non-winter months, ratio of rates and relative change in winter:non-winter ratios for potential modifying factors; 1996–2000

	Rate per 10,000 person years (No of deaths)*		**Winter:non-winter ratio (95% CI)‡ Unadjusted**	**Winter:non-winter ratio relative to baseline category§ (95% CI)‡**	**p-value**
	**Winter**	**Non-winter**			

**All**	9.4 (5762)	5.5 (6178)			

**Age (years):**					
0–4	0.9 (42)	0.4 (35)	2.40 (1.31, 4.41)	1.00	
5–14	0.0 (3)	0.0 (4)	1.50 (0.34, 6.58)	0.61 (0.13, 2.96)	
15–29	0.1 (16)	0.1 (26)	1.24 (0.62, 2.45)	0.50 (0.22, 1.11)	
30–59	1.1 (260)	0.7 (348)	1.49 (1.01, 2.18)	0.61 (0.35, 1.06)	
60–79	29.8 (2381)	17.5 (2796)	1.61 (1.31, 1.97)	0.67 (0.40, 1.11)	
80+	180.2 (3060)	103.6 (3509)	1.66 (1.40, 1.97)	0.67 (0.40, 1.13)	p = .56
**Gender:**					
Male	9.4 (2930)	5.9 (3501)	1.64 (1.40, 1.92)	1.00	
Female	9.4 (2932)	5.2 (3217)	1.74 (1.49, 2.03)	1.15 (0.99, 1.34)	p = .08
**Ethnicity**†:					
Non-Maori	9.0 (5328)	5.2 (6159)	1.68 (1.39, 2.02)	1.00	
Maori	15.7 (434)	9.9 (559)	1.58 (1.08, 2.33)	0.90 (0.72, 1.12)	p = .35
**NZDep96:**					
1–3	7.5 (1233)	4.6 (1504)	1.49 (1.10, 2.03)	1.00	
4–6	9.0 (1662)	5.3 (1948)	1.64 (1.23, 2.20)	1.13 (0.93, 1.37)	
7–10	10.9 (2867)	6.2 (3266)	1.80 (1.38, 2.35)	1.12 (0.93, 1.36)	p = .40
**RHA:**					
North	8.5 (1790)	4.8 (2012)	1.91 (1.37, 2.64)	1.00	
Midland	9.6 (1165)	5.6 (1369)	1.52 (1.08, 2.14)	0.87 (0.70, 1.08)	
Central	9.5 (1416)	5.8 (1728)	1.49 (1.06, 2.10)	0.95 (0.77, 1.17)	
South	10.6 (1391)	6.2 (1609)	1.71 (1.21, 2.42)	0.95 (0.77, 1.17)	p = .67

* No of deaths in winter correspond to a 4 month period; non-winter, an 8 month period

‡ Generalised negative binomial regression model; overdispersion a linear combination of age group and ethnicity

§ Adjusted for all other covariates including covariate by winter interaction terms

† Mortality rates have been age-standardised to account for different population structures of Maori and Non-Maori

The mortality rate for diseases of the circulatory system during winter was 33.7 per 10,000 person years over this 5-year period; 24% higher than expected from non-winter rates (Table 4). Almost eighty percent of mortality from diseases of the circulatory system was caused by IHD (56%) and CVD (23%). The winter:non-winter mortality ratio for IHD over this five-year period was 1.17 (95% CI 1.13, 1.21) and for CVD it was 1.19 (95% CI 1.13, 1.26). After adjusting for all other covariates in the regression model, estimates of the magnitude of winter:non-winter mortality from diseases of the circulatory system were around 40% higher in those aged sixty plus than in those aged under 30 at the time of death. There was no evidence to suggest that winter:non-winter circulatory disease MRRs differed by gender, prioritised ethnicity, NZDep or RHA.

**Table 4:** Rates of mortality from diseases of the circulatory system in winter and non-winter months, ratio of rates and relative change in winter:non-winter ratios for potential modifying factors; 1996–2000

	Rate per 10,000 person years (No of deaths)*		**Winter:non-winter ratio (95% CI)‡ Unadjusted**	**Winter:non-winter ratio relative to baseline category§ (95% CI)‡**	** p-value**
	**Winter**	**Non-winter**			

**All**	33.7 (20664)	27.4 (33576)	1.24 (1.13, 1.36)		

**Age (years):**					
0–4	0.1 (3)	0.2 (18)	0.33 (.09, 1.22)	1.00†	
5–14	0.1 (6)	0.0 (8)	1.50 (0.52, 4.31)		
15–29	0.4 (50)	0.4 (112)	0.89 (0.62, 1.27)		
30–59	6.8 (1678)	6.2 (3065)	1.05 (0.76, 1.46)	1.30 (0.90, 1.90)	
60–79	100.7 (8057)	83.1 (13264)	1.16 (0.95, 1.40)	1.40 (1.00, 1.96)	
80+	640.2 (10870)	505.1 (17109)	1.25 (1.13, 1.37)	1.44 (0.99, 2.08)	p = .22
**Gender:**				
Male	33.8 (10148)	28.0 (16773)	1.28 (1.13, 1.46)	1.00	
Female	33.6 (10516)	26.9 (16803)	1.21 (1.06, 1.37)	1.06 (0.94, 1.19)	p = .33
**Ethnicity^:**				
Non-Maori	32.3 (19071)	26.2 (30797)	1.27 (1.15, 1.40)	1.00	
Maori	55.8 (1593)	46.7 (2779)	1.18 (0.96, 1.44)	0.93 (0.81, 1.07)	p = .32
**NZDep96:**					
1–3	28.7 (4742)	23.5 (7733)	1.20 (1.01, 1.42)	1.00	
4–6	34.2 (6325)	27.9 (10292)	1.23 (1.05, 1.45)	1.07 (0.92, 1.24)	
7–10	36.4 (9597)	29.6 (15551)	1.28 (1.10, 1.49)	1.04 (0.90, 1.20)	p = .67
**RHA:**					
North	29.1 (6132)	23.7 (9997)	1.24 (1.03, 1.49)	1.00	
Midland	34.4 (4200)	27.7 (6754)	1.34 (1.11, 1.62)	0.97 (0.83, 1.15)	
Central	35.7 (5338)	28.7 (8563)	1.17 (0.97, 1.42)	1.00 (0.85, 1.18)	
South	38.2 (4994)	31.7 (8262)	1.21 (1.00, 1.48)	0.96 (0.81, 1.13)	p = .93

* No of deaths in winter correspond to a 4 month period; non-winter, an 8 month period

‡ Generalised negative binomial regression model; overdispersion a linear combination of age group and ethnicity

§ Adjusted for all other covariates including covariate by winter interaction terms

† Because of small cell counts, age group 0–29 years was used as the baseline category

^ Mortality rates have been age-standardised to account for different population structures of Maori and Non-Maori

## Discussion

Winter mortality rates in NZ from 1980 to 2000 were 18% higher than expected from non-winter rates; this is 2% higher than the mean for 14 European countries [8]. NZ appears to have similar EWM to the UK with New Zealanders more vulnerable to EWM than Scandinavians. We could not find recent estimates of EWM for other Southern Hemisphere countries, although analysis of 1976–1984 mortality data indicated NZ had more extreme seasonal mortality than Australia and 11 other countries in the Southern Hemisphere [4,22].

Of the factors thought to indicate vulnerability to winter death, age was the only clear predictor. Patterns of EWM by age group followed similar trends to mortality rates with the young and the elderly particularly vulnerable. In the older age groups the seasonal increase rose steeply with age. EWM from respiratory disease was particularly striking in under-five year olds.

Females may be more vulnerable than males to winter mortality. After adjusting for all major covariates the winter:non-winter MRR for females was 9% higher than for males. There was no evidence to suggest that patterns of EWM differed by ethnicity.

Although the lack of seasonal variation by region is consistent with a number of studies, use of 4 RHAs is a relatively insensitive way of assessing the impact of geography on seasonal mortality, with climate within regions varying considerably [4,6]. It would be interesting to compare EWM between smaller regions and to explore whether urban-rural differences exist.

Studies of variation of seasonal mortality by socioeconomic status continue to provide conflicting results. Using a small-area level measure of deprivation this research found no evidence suggesting that people from more deprived areas were particularly vulnerable to EWM. One assumption made when calculating the NZDep decile associated with a mortality event is that the individual's last known usual address is applicable. Future research using individual-level socioeconomic variables would provide interesting comparisons.

Mortality from respiratory disease was strongly influenced by season, with winter estimates 66% higher than expected from non-winter rates. Although the winter:non-winter MRR for circulatory disease was lower at 24%, mortality from this category had the largest absolute fluctuations between seasons as mortality from circulatory diseases comprised 40% of total mortality compared to 10% from respiratory disease. Although certain infectious and parasitic diseases had a strong seasonal pattern, this grouping contributes only 0.5% of total mortality in NZ. Analysis using ICD chapters has many limitations as these categories are broad and not based strictly on aetiology. For example, a previous analysis of NZ deaths with an infectious disease cause found that only 10% of those recorded from 1988–2000 were coded to the ICD-9 infectious disease chapter [23].

Because numerator data were obtained from a difference source (NZHIS) than population denominators (SNZ), there are potential errors with calculating rates of mortality between years and population subgroups. However, comparisons between winter rates and non-winter rates should be relatively robust.

The lack of evidence to confirm the role of factors thought to indicate vulnerability to winter death may have been due to limitations in the measurement of factors such as ethnicity and deprivation at the area level. Statistical power may also have been an issue with some of the interaction terms that we examined for evidence of differential effects on EWM, but the 137,583 deaths used in the detailed 1996–2000 analysis would generally not be considered a small dataset.

A major limitation of the modelling approach taken is the implied sudden change in mortality rate at the start and end of winter. Use of a continuous method to model the pattern of seasonal change would offer a more realistic approach. Fourier analysis could be used to fit a sinusoidal wave to monthly mortality data and from this model estimates of absolute and relative differences between peak mortality and mortality at the nadir could be obtained.

Exploring the role that meteorological variables (e.g. temperature, humidity) and other seasonal exposures (e.g. influenza, air pollution) play in EWM was beyond the scope of this paper. Further research should explore whether more tailored conclusions could be obtained if the specific pathways driving EWM were identified. For example, if annual peaks in winter mortality were associated with particularly cold indoor temperatures then it would be sensible to place greater emphasis on improved home heating and insulation whereas if winter peaks were associated with influenza epidemics then greater emphasis might be placed on more comprehensive influenza vaccination programme [24]. Future research could also investigate the effects of other potentially modifiable seasonal factors such as indoor air pollution, household crowding, and vitamin D deficiency.

## Conclusion

EWM in NZ is substantial and at the upper end of the range observed internationally. Interventions to reduce EWM are important, but the surprising lack of variation in EWM by ethnicity, region and deprivation, provides little guidance for how such mortality can be reduced. More targeted research is needed to explore a number of other possible factors that could be contributing to EWM, including the role of climate, influenza, behaviour, crowding in winter, levels of home heating & thermal performance of houses.

## Competing interests

The author(s) declare that they have no competing interests.

## Authors' contributions

GD performed the statistical analysis and drafted the manuscript. MB conceived of the study, participated in its design and provided advice based on considerable knowledge of the subject area. SH participated in the design of the study, the acquisition of data and the interpretation of results. JC provided substantive biostatistical advice and helped interpret the data. All authors revised drafts of the manuscript and read and approved the final manuscript.

## Funding

University of Otago Research Grant

## Pre-publication history

The pre-publication history for this paper can be accessed here:


